# ﻿*Liparistianchiensis* (Orchidaceae), a new species from Gansu, China

**DOI:** 10.3897/phytokeys.219.90351

**Published:** 2023-01-23

**Authors:** Xiao-Juan Liu, Xue-Gang Sun

**Affiliations:** 1 College of Forestry, Gansu Agricultural University, Lanzhou 730070, Gansu, China Gansu Agricultural University Lanzhou China

**Keywords:** Malaxideae, morphology, new species, Wenxian County

## Abstract

*Liparistianchiensis* (Orchidaceae, Epidendroideae), a new species from Wenxian County, Gansu Province, China, is described and illustrated, based on morphological characters. *Liparistianchiensis* is morphologically similar to *L.damingshanensis*, *L.pauliana* and *L.mengziensis* with erect, lax flowered-inflorescences, small persistent floral bracts, small greenish-purple flowers, spreading sepals, free reflexed and linear petals, a lip with 2 calli near the base and an arcuate column. *Liparistianchiensis* differs from *L.pauliana* by the single and much smaller leaf, shorter sepals and petals, smaller and reflexed oblong lip. It differs from *L.mengziensis* by having fewer and larger flowers and not connate lip apex. The novelty mostly resembles *L.damingshanensis*, but can be readily identified by having longer sepals and a reflexed oblong lip. *Liparistianchiensis* only occurs in evergreen broad-leaved forest around a mountain lake in Wenxian County, Gansu Province, China.

## ﻿Introduction

The genus *Liparis*Richard (1817: 21) belongs to tribe Malaxideae (subtribe Malaxidinae) of the subfamily Epidendroideae (Chase et al. 2015) and comprises approximately 424 species (Plants of the World Online (https://powo.science.kew.org/)) with cosmopolitan distribution from the tropics and subtropics to the temperate and alpine regions. Most species in this genus occur in Southeast Asia as well as in Africa, Australia and the Americas (Pridgeon et al. 2005). *Liparis* is easily distinguished from its allies, *Malaxis* Sol. ex Sw. (Swartz 1788: 119), *Oberonia*Lindley (1830: 15) and *Hippeophyllum* Schltr. in Schumann and Lauterbach (1905: 107), based on its floral morphology (viz. resupinate flowers with narrow linear petals and a curved, slightly winged column lacking a foot) (Pearce and Cribb 2002; Chen et al. 2009). The taxonomy of the genus is very confused and inconsistent (Margońska and Szlachetko 2001, 2004; Pridgeon et al. 2005; Liu et al. 2008) and some research indicated that *Liparis* were polyphyletic. Tang et al. (2015) performed molecular and morphological analyses to establish the phylogenetic relationships within Malaxideae, the results supporting the division of *Liparis* into 11 genera, but the intergeneric relationships remain unclear and the genus definition is considerably controversial. Therefore, we tentatively opted to maintain *Liparis* as a broad concept for the present taxonomic treatment. There are approximately 70 species of *Liparis* in China, including many recently described taxa (see Chen et al. (2009); Feng and Jin (2010); Jin (2010, 2011); Wu et al. (2012); Hsu (2013); Li and Yan (2013); Su et al. (2014); Su et al. (2015); Tang et al. (2015); Li et al. (2019); Ya et al. (2021)).

Wenxian County, Gansu Province, is situated in the northwest of China, in a transitional zone between the north subtropical and warm temperate regions. The altitudes vary from 595 to 4072 m. Variations in climate combined with complex topography have resulted in high plant diversity (Li 2014). Although being a biodiversity hotspot in China, the flora of this area is still unknown despite many years of directed fieldwork and research. Several new species have been described in the last decades, such as *Cardaminetianqingiae* Al-Shehbaz & Boufford in Ihsan (2008: 89) (Brassicaceae), *Spiranthessunii* Boufford & Wen H.Zhang (2008: 261) (Orchidaceae), *Vitiswenxianensis* W.T.Wang (2010: 288) (Vitaceae), *Clematisaustrogansuensis* W.T.Wang & L.Q.Li (2011: 285) (Ranunculaceae) and, more recently, *Oreochariswenxianensis* XiaoJ.Liu & X.G.Sun (2021: 182) (Gesneriaceae) and *Saxifragasunhangiana* T.Deng, X.J.Zhang & J.T.Chen in Zhang et al. (2022: 197) (Saxifragaceae).

Tianchi Lake in Wenxian County is a famous mountain lake in China, covering an area of nearly 1 km^2^, surrounded by mountains, rich in plant diversity. During our field trip around Tianchi Lake in 2021, we collected nearly 20 orchid species, including an unknown *Liparis*. Further examination by means of morphological comparison indicated that it represented a new species, which is described and illustrated here.

## ﻿Material and method

Specimens from the single known subpopulation of the putative new species were collected during field expeditions in 2021. Morphological characters of five living plants, including three flowering and two non-flowering individuals, were observed, measured and photographed under an Olympus stereozoom microscope. Specimens were deposited in the Forestry Herbarium of Gansu Agricultural University (**GAUF**), located in Lanzhou City, Gansu Province, China.

After consulting relevant literature (Pearce and Cribb 2002; Pridgeon et al. 2005; Liu et al. 2008; Chen et al. 2009; Yang et al. 2009; Feng and Jin 2010; Jin 2010, 2011; Wu et al. 2012; Hsu 2013; Li and Yan 2013; Su et al. 2014; Su et al. 2015; Tang et al. 2015; Li et al. 2019; Ya et al. 2021) and examining scans of type specimens and other relevant herbarium specimens of *Liparis* available online from AMES, BM, E, L, K, P and PE (acronyms following Thiers, continuously updated), JSTOR Global Plants (http://plants.jstor.org), China National Specimen Information Infrastructure NSII (http://www.nsii.org.cn/2017/home-en.php) and The Chinese Virtual Herbarium NPSRC (http://www.cvh.ac.cn), the taxonomic status of the putative new species and morphologically similar species were investigated.

## ﻿Taxonomic treatment

### ﻿ Liparis tianchiensis

Taxon classificationPlantaeScorpaeniformesLiparidae

X.J.Liu & X.G.Sun sp. nov.

5514B259-1E37-5236-B1E1-5F7B29DBAC7A

urn:lsid:ipni.org:names:77312597-1

Figs 1 , 2

#### Type.

China. Gansu Province, Longnan City, Wenxian County, Tianchi Lake, growing on moss in evergreen broad-leaved forest, elev. 1680 m, 15 July 2021, X.J. Liu & H. Lin, WX20210715001 (holotype: GAUF!, isotype: GAUF!).

#### Diagnosis.

*Liparistianchiensis* is similar to *L.damingshanensis* with single small leaf, erect, lax flowered-inflorescences, small greenish purple flowers, spreading sepals, free reflexed and linear petals, a lip with 2 calli near the base and an arcuate column. It, however, differs from the latter by having a longer dorsal sepal (9.0–11.0 mm vs 6.0–8.0 mm) and a longer (8.0–10.0 vs 5.0–7.0 mm) and oblong (vs obovate-triangular) lip.

#### Description.

Terrestrial herbs. Pseudobulbs ovoid or oblong-ovoid, 0.5–1.5 cm long, 0.4–1.0 cm in diameter, covered with white membranous remnant sheaths. Leaf single, green, glabrous, petiole sheath like, 0.5–1.0 cm long, amplexicaul, not articulate; blade ovate, 1.2–3.0 cm × 1.0–2.0 cm, contracted and decurrent into petiole at base and acute or obtuse at apex, margin entire and flat or slightly undulate. Inflorescence 4.0–9.0 cm long; peduncle slightly compressed cylindrical, narrowly winged on either side; rachis laxly 2–4-flowered; floral bracts ovate-triangular, 2.0–3.0 mm long, membranous, greenish-white. Flowers fully opening, greenish-purple, 1.6–2.2 cm across; pedicel and ovary greenish-purple, twisted, 1.0–1.2 cm long. Dorsal sepal ovate-oblong, margins often revolute, apex obtuse, 9.0–11.0 mm × ca. 1.0 mm, translucent greenish-white. Lateral sepals parallel under the lip, obliquely ovate-elliptic, margins often revolute, apex obtuse, 8.0–11.0 mm × ca. 1.0 mm, translucent greenish-white. Petals deflexed, narrowly oblong-falcate, margins revolute, 8.0–10.0 mm × 0.3–0.5 mm, purple. Lip oblong, 8.0–10.0 mm × 3.0–5.0 mm, pale purple, with 2 short longitudinal triangular lamellae at the shallowly concave base, mucronate at apex; mid-vein dull-purple, broad and stout, conspicuous from the base up to the apex. Column conspicuously incurved, 4.0–5.0 mm long, ca. 1 mm in diameter, apex with 2 short, triangular wings, base purple and swollen; anther-cap obovate, glabrous; pollinia four in two pairs, yellow. Fruit not observed.

#### Etymology.

The specific epithet is derived from the type locality Tianchi Lake, Wenxian County, Gansu Province, China.

#### Distribution and habitat.

*Liparistianchiensis* is terrestrial and grows in shaded and damp moss-covered areas in evergreen broad-leaved forest, forming scattered colonies on the slopes of Tianchi Lake at 1680 m a.s.l. For the time being, *Liparistianchiensis* is only known from the type locality. The subpopulation is small, with less than 200 individuals.

**Figure 1.**
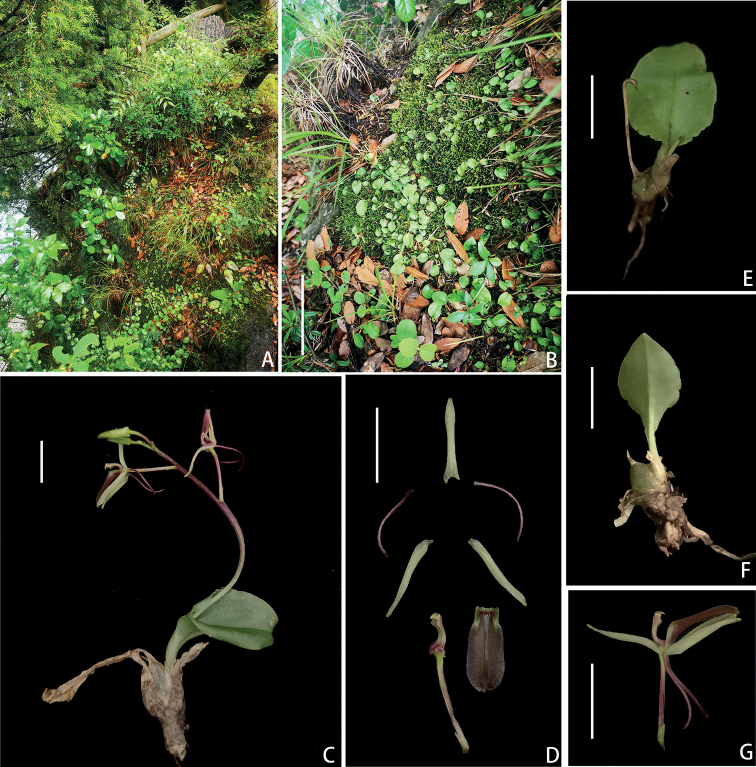
*Liparistianchiensis* sp. nov. **A** habit **B** non-flowering plants *in situ***C** flowering individual **D** dissected floral parts **E** leaf, adaxial view **F** leaf, abaxial view **G** flower, lateral view. Scale bars: 10 cm (**B**); 1 cm (**C–G**).

**Figure 2.**
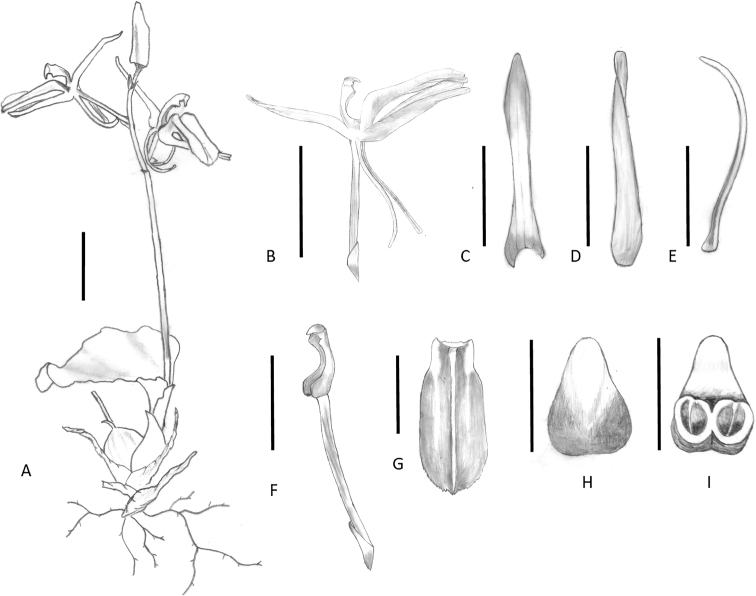
*Liparistianchiensis* sp. nov. **A** individual in bloom **B** flower, lateral view **C** dorsal sepal **D** lateral sepal **E** petal **F** pedicel, ovary and column **G** lip **H** anther-cap, abaxial view **I** anther-cap, adaxial view. Scale bars: 1 cm (**A, B**); 0.5 cm (**C–G**); 1 mm (**H, I**). Drawn by Hui Lin from the holotype.

#### Flowering phenology.

June and July.

### ﻿Key to *Liparistianchiensis* and its related species

**Table:** 

1	Pedicel and ovary shorter than 1.0 cm, lip apex connate along the margins	***Liparismengziensis***
–	Pedicel and ovary longer than 1.0 cm, lip apex flat	**2**
2	Flower size larger than 1.0 cm, leaf 2, very rarely 1	***Liparispauliana***
–	Flower size not larger than 1.0 cm, leaf 1	**3**
3	Lip obovate-triangular, 5.0–7.0 × 5.0–6.0 mm, dorsal sepal 6.0–8.0 mm long	***Liparisdamingshanensis***
–	Lip oblong, 8.0–10.0 × 3.0–5.0 mm, dorsal sepal 9.0–11.0 mm long	***Liparistianchiensis***

## Supplementary Material

XML Treatment for Liparis tianchiensis
